# Cortical activation and functional connectivity during different attention tasks using functional near-infrared spectroscopy in middle-aged and elderly people

**DOI:** 10.1186/s40001-025-02597-1

**Published:** 2025-05-05

**Authors:** Lijuan Ding, Yiru Zhang, Youshu Xie, Yongzhi He, Yunyun Wang, Jiachun Lu, Rizhao Pang, Wenchun Wang, Zhesi Chen

**Affiliations:** 1 Department of Rehabilitation, the General Hospital of Western Theater Command (Affiliated Hospital of Southwest Jiaotong University), College of Medicine, Southwest Jiaotong University, Chengdu, China; 2 Jinchen Rehabilitation Hospital of Chengdu, Chengdu, China; 3 Department of Occupational Therapy, Guangdong Work Injury Rehabilitation Hospital, Guangzhou, China; 4 North Sichuan Medical College, Nanchong, China; 5 Department of Rehabilitation, The Second Affiliated Hospital, Guangzhou Medical University, Guangzhou, China; 6 Chengdu Eighth People’s Hospital (Geriatric Hospital of Chengdu Medical College), Chengdu, China

**Keywords:** Cortical activation, Functional connectivity, Attention, FNIRS

## Abstract

**Background:**

Attention plays a vital part in the cognitive process, where different kinds of attention are associated with separate brain mechanisms. The objective of this research was to investigate the patterns of brain activation and functional connectivity in middle-aged and elderly individuals, while they were engaged in various attentional tasks, with the intention of establishing a reference foundation for the clinical treatment of attention disorders.

**Materials and methods:**

A total of 44 healthy middle-aged and elderly persons (47.1% women) aged over 40 were enrolled in this study. The digital cancellation test (DCT), the paced auditory serial addition test (PASAT), the Stroop colour-word test, and the trail making test (TMT) are, respectively, associated with four types of attention tasks: sustained attention, divided attention, selective attention, and attention shifting. Functional near-infrared spectroscopic imaging was employed to measure the concentration of brain oxyhaemoglobin in the subjects, while they were performing these four attention tasks.

**Results:**

In this study, we found distinct activation patterns in brain areas, such as BA-3, BA-4, BA-6, and others. Functional connectivity analysis revealed that the frontal and right parietal lobes consistently showed higher density and strength of connections across tasks, with the PASAT task exhibiting the highest number of connections exceeding the threshold. Notably, the DCT task demonstrated significant correlations in oxygen fluctuations among several brain regions, while the TMT-B task highlighted strong functional connectivity within the bilateral frontal and parietal lobes.

**Conclusions:**

This research provides evidence that middle-aged and elderly people have different brain activation and functional connectivity patterns in different attentional tasks, suggesting individualized treatment for attention disorder patients based on impairment type and location.

*Trial registration*: This study has been registered through the Chinese Clinical Trial Registry (ChiCTR2400087755).

## Introduction

Human beings possess a finite capacity to process information from the external environment, necessitating the strategic deployment of attention to allocate cognitive resources effectively [1]. Attention encompasses the capacity of human mental activity to concentrate on specific stimuli, serving as the foundation for the allocation of cognitive resources and the execution of various cognitive functions [2]. While Posner and Petersen's tripartite framework (alerting, orienting, and executive control) has dominated attention research for three decades [3], several limitations remain. Specifically, neuroimaging studies on attention have focused on single-task paradigms. Most attention studies have focused on children and attention–deficit hyperactivity disorder (ADHD), and no study has simultaneously measured cortical activation patterns and functional connectivity across different types of attention in aging populations. This gap is particularly significant given the brain cortex's structural decline with aging, which directly impacts attentional control capacity. Consequently, it is imperative to investigate the cortical underpinnings of these functions to glean insights that can inform future research and therapeutic approaches.

Functional near-infrared spectroscopy (fNIRS) is a novel non-invasive brain function imaging technique, which has been widely used in cognitive neuroscience, social neuroscience, clinical neuroscience, etc., because of its good temporal and spatial resolution, as well as small size and mobility [4]. The principle relies on the use of near-infrared light, which penetrates biological tissues effectively, with the fibre optic probe launching the near-infrared light to penetrate the cerebral cortex and, through the scattering, diffuse reflection to the receiving probe, by the light absorption changes to monitor the local oxyhaemoglobin (oxy-Hb) and deoxyhaemoglobin (deoxy-Hb) concentration changes as most of the biological tissues in the 650–950 nm near-infrared light and the concentration changes [5]. A very famous hypothesis was formulated by Roy and Sherrington in 1890:"Brain blood supply locally corresponds to local changes in functional activity"[6]. As the cortex responds, fNIRS can monitor the changes in oxy-Hb and deoxy-Hb and respond to the functional state of the brain.

Pinti pointed out that the dynamics of the relationship between oxyhaemoglobin and deoxyhaemoglobin signals in different brain regions and across different tasks and conditions remains largely to be investigated [7]. This fits in with the purpose of this study. The brain activity and functional connectivity in middle-aged and elderly people during the performance of different attention tasks are not known. In this present study, fNIRS was used to detect brain activity and functional connectivity in middle-aged and elderly people during different attentional tasks, which will serve as a reference and basis for future studies.

## Materials and methods

### Study design, participants

This is an observational study with a cross-sectional design. The whole process references the *STROBE statement *(https://www.strobe-statement.org), and the experimental protocol was approved by the General Hospital of Western Theater Command Human Research Ethics Committee. This study has been registered through the Chinese Clinical Trial Registry (ChiCTR2400087755).

The study was conducted at the General Hospital of Western Theater Command. Inclusion criteria: (a) ages over 40 years; (b) primary school degree or above, able to co-operate with the experiment; and (c) right-handed. Before the experiment, each subject learned about the experimental procedure and signed an informed consent agreement. All subjects were from family members of hospital inpatients, carers, or hospital staff. Subjects with traumatic brain injury, dementia, stroke, psychiatric disorders, or other disorders that may cause cognitive impairment were excluded.

### Experimental procedure

The test was conducted in a separate, quiet room. Only the subject and the tester are present during the test to avoid other distractions. The subject was seated in a comfortable chair, and a table for completing tasks was in front of the chair. It was clear that the subject understood and knew the rules before each task. The experimental protocol consisted of four phases: (a) demographic information survey and cognitive function assessment; (b) scales assessment; and (c) fNIRS acquisition under different attention tasks. The attention tasks were derived from four clinically used attention assessment scales, and subjects performed the fNIRS collection while performing the attention tasks.

The Mini-Mental Status Examination (MMSE) was used to assess the cognitive functioning of the subjects, and the scale covers 6 areas of orientation, memory, attention, numeracy language skills, and visuospatial cognitive skills with a total of 30 points. Each item was scored 1 point for a correct response and 0 points for an incorrect or do not know. A score of less than 27 suggests cognitive impairment. The digital span test (DST) is a common method of checking the attention span. The examiner names a series of numbers and asks the subject to repeat them forward and backward, and the highest number of correctly repeated numbers is the subject's numerical distance, i.e., the score obtained. The test is strongly influenced by memory.

### Attention tasks

The Digit Cancellation Test (DCT) measures sustained attention by having subjects cancel out a specific digit within a set time and counting correct cancellations [8]. Normal performance is defined as omitting 0–2 digits in 120 s. The Attention Persistence Index is calculated as (total words accessed/cancel time) * (number of cancellations due/number of correct cancellations–number of incorrect cancellations). The Stroop Colour-Word Test assesses selective attention across three steps: reading colour names (Stroop A), naming colours (Stroop B), and naming colours of colour-word pairs (Stroop C) [9]. The interference effect is measured by SIE-time (Stroop C time–Stroop B time) and SIE-correct (Stroop B correct–Stroop C correct), with larger values indicating weaker interference suppression. The Trail Making Test (TMT) evaluates attentional shifting, with Part A requiring number sequencing and Part B requiring alternating between two colours to connect numbers [10]. The TMT interference is scored as the time difference between TMT-B and TMT-A. Finally, the Paced Auditory Serial Addition Test (PASAT) tests attention distribution by asking subjects to add pairs of numbers presented auditorily with a maximum score of 60 points [11].

### NIRS data acquisition

**Fig. 1:**
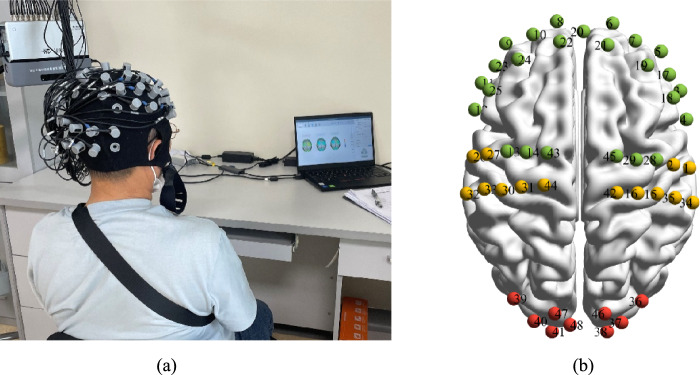
**a** Participant is being tested (**b**) each sphere represents a channel, with the number denoting the channel number. The green spheres span the frontal lobe, the yellow spheres are associated with the parietal lobe, and the red ones pertain to the occipital lobe

**Fig. 2:**
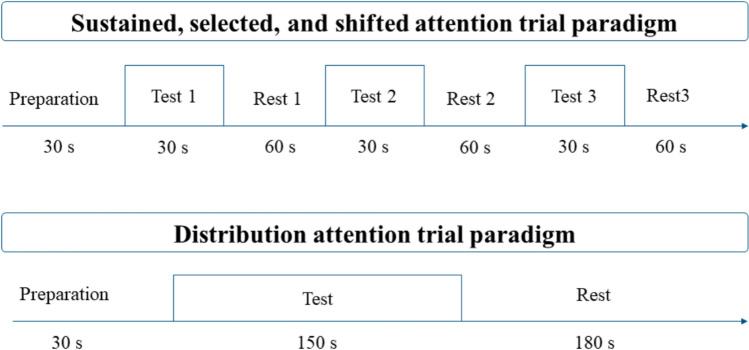
Experimental protocol

For the collection of cerebral blood oxygen signals, a portable near-infrared spectroscopy (NIRS) device with 48 channels (comprising 24 laser emitters and 16 detectors), manufactured by Danyang Huichuang Medical Equipment Co., Ltd. in China, was employed. This device was configured to cover multiple brain regions, namely, the frontal, temporal, parietal, and occipital lobes, following the international 10/20 system. The detailed distribution of the channels is depicted in Fig. 1b through the utilization of BrainNet Viewer software [12]. The system was equipped with two wavelengths of near-infrared light, specifically 730 nm and 850 nm, which enabled the detection of alterations in haemoglobin concentration within the cerebral cortex. The sampling frequency was established at 11 Hz. The NIRS experiment adopted a block design, wherein a 30-s resting preparation phase preceded each task. The sustained, selective, and shifted attention tasks consisted of 1 block, each with a 30-s task period followed by a 60-s resting period, amounting to a total of 3 blocks. The divided attention task block comprised a 150-s task period and a 180-s resting period (Fig. 2).

### Data pre-processing

The collected data were pre-processed using the NirSpark software package (Danyang Huichuang, China), and the processing steps were: (i) inspecting the data quality by the data quality analysis tool and removing poor quality data (CV > 20, CV = std signal/mean signal*100); (ii) removing time intervals and motion trajectories unrelated to the experiment using moving standard deviation and spline interpolation [13]; (iii) applying 0.01–0.08 Hz Butterworth bandpass filtering on physiological noise and interfering signals to retain signals in the frequency domain; and (iv) converting the raw optical signals to blood oxygen concentration.

### Data analysis

Considering that oxy-Hb exhibits a higher sensitivity to cerebral blood flow changes than deoxy-Hb and has a better signal-to-noise ratio and remeasurement reliability [14–16], we chose oxy-Hb for further statistical analysis. The features were derived by computing the block averages across all channels, which included the mean and integral of oxy-Hb during the task period, the centroid within the block, and the initial slope of oxy-Hb at the task's onset. Subsequently, these features were extracted and subjected to normality testing using the Shapiro–Wilk test. The activated channels'features were then analysed using *t* tests for parametric data or non-parametric tests, as appropriate, employing SPSS 25.0 software (IBM Corp., NY, USA).

Functional connectivity was analysed by performing Spearman`s correlation between the time series of each channel-to-channel pair after being converted to z by the formula (*z* = 1/2(ln[(1 + *ρ*)/(1 − *ρ*)]), which was conducted using NirSpark. A *p* value threshold of < 0.05 was considered statistically significant. All statistical tests were two-tailed, with multiple comparisons across channels being corrected using the false discovery rate (FDR) via the Benjamini–Hochberg (BH) procedure [17]. The outcomes were regulated using a threshold value of 0.5 to enable enhanced comparability, as this threshold is conventionally associated with medium correlation strength in statistical analyses [18]. The two predominant models for examining attention networks are the tripartite subsystem model put forth by Posner and Petersen in the early 1990 s [3] and the dual network model proposed by Corbetta and Shulman [19]. Referring to these two seminal models and the particularities of this research, the region of interest (ROI) was delineated, as illustrated in Table 1.

**Table 1:** Corresponding region of interest to channels and the Brodmann area

ROI	Channels	Brodmann area
The left dorsolateral prefrontal cortex (DLPFC.L)	9, 22, 24	9, 46
The right dorsolateral prefrontal cortex (DLPFC.R)	5, 19, 21	9, 46
The left primary motor cortex (M1.L)	26, 31, 44	4
The right primary motor cortex (M1.R)	2, 15, 16, 42	4
The left prefrontal cortex (PFC.L)	13, 14, 27, 43	6
The right prefrontal cortex (PFC.R)	28, 29, 45	6
The left primary somatosensory cortex (S1.L)	30, 33	3
The right primary somatosensory cortex (S1.R)	1, 35	1
The superior frontal gyrus, medial (SFG.Med)	20	10
The left superior frontal gyrus (SFG.L)	8, 10	10
The right superior frontal gyrus (SFG.R)	6, 7	10
The left temporoparietal junction (TPJ.L)	32	2
The right temporoparietal junction (TPJ.R)	34	40
The left primary visual cortex (V1.L)	40, 41, 48	17
The right primary visual cortex (V1.R)	37, 38	17
The left secondary visual cortex (V2.L)	47	18
The right secondary visual cortex (V2.R)	46	18
The left visual association cortex (V3.L)	39	19
The right visual association cortex (V3.R)	36	19
The left ventral frontal cortex (VFC.L)	11, 12, 23, 25	45
The right ventral frontal cortex (VFC.R)	3, 4, 17, 18	45

## Results

### Demographics and clinical characteristics

**Fig. 3:**
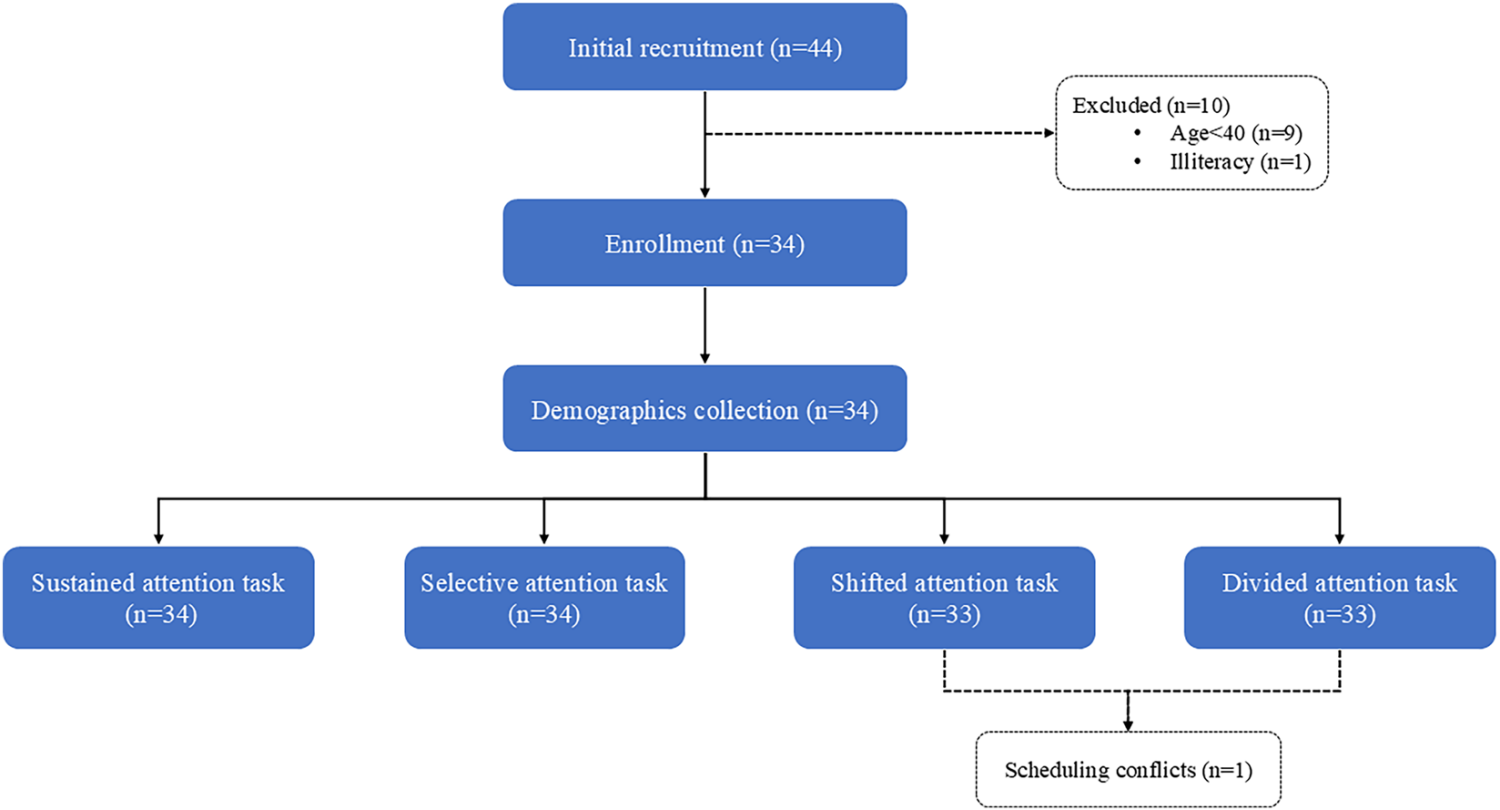
Registration flowchart

The demographics and clinical features of the subjects are summarized in Table 2. A total of 44 subjects were enrolled from June to July 2024, with 34 subjects (age range 40–65 years; mean age 54.26; 18 males and 16 females) ultimately participating in this study (Fig. 3). One participant withdrew due to unforeseen circumstances and failed to complete two attention tasks. The educational background of participants predominantly consisted of secondary education, accounting for the majority (*n* = 18). Participants demonstrated preserved overall cognitive function, as evidenced by a mean MMSE score of 27.68 and an average digit span test score of 12.

**Table 2:** Demographics, clinical, and task performance measures

	Mean (SD)/median (IQR)	*N* (%)
Age (y)	54.26 (5.98)	
MMSE	27.68 (1.63)	
DST	12 (2)	
DCT-time (s)	53.71 (12.72)	
DCT-correct	19 (1)	
DCT-index	4.56 (1.5)	
Stroop C-time (s)	87 (27.48)	
Stroop C-correct	46 (7.75)	
SIE-time	32(23.5)	
SIE-correct	1.5(6.75)	
TMT B-time (s)	84 (48)	
PASAT	31.48 (10.28)	
Gender		
Male		18 (52.9)
Female		16 (47.1)
Education		
Primary school		12 (35.3)
Middle school		18 (52.9)
Bachelor's degree and above		4 (11.8)

### Cortical activations

A one-sample *t* test or rank sum test for each feature, we concluded which channels were significantly activated when executing different attention tasks (Table 3). The mean concentration of oxy-Hb and the integral values over the task period were employed as metrics to determine the activation area and its intensity. We found that the main activation sites in the cerebral cortex were concentrated in the left primary somatosensory cortex (BA-3), the left primary motor cortex (Brodmann Area (BA)-4), the pre-motor and supplementary motor cortex (BA-6), the frontopolar area (BA-10), the primary visual cortex (V1) (BA-17), the left secondary visual cortex (V2) (BA-18), the visual association cortex (V3) (BA-19), the pars of Broca's area (BA-44, 45), and the dorsolateral prefrontal cortex (BA−46) during sustained attention task (DCT); the major activation sites were the left BA-4, the right BA-6, and the BA-17 activated when subjects were performing divided attention task (PASAT); the right primary somatosensory cortex (BA-1, 3), the BA-4, the right BA-6, 18, 19, 44, and the left BA-10,17,45,46 activated when performing a selective attention task (Stroop C); the BA-4, 6, 17, 46, and the left BA-10, 18, 19, 44, 45 activated when performing a shifted attention task (TMT-B).

**Table 3:** Features and activated channels of different attention tasks

Tasks	Features	Activated channels	Brodmann areas
DCT	Mean	3, 4, 5, 7, 8, 9, 11, 12, 19, 20, 24, 26, 27, 30, 33, 36, 37, 38, 39, 41, 46, 47, 48	3, 4, 6, 10, 17, 18, 19, 44, 45, 46
	Integral	3, 4, 5, 7, 8, 9, 11, 12, 19, 20, 24, 26, 27, 30, 33, 36, 37, 38, 39, 41, 47, 48	3, 4, 6, 10, 17, 18, 19, 44, 45, 46
	Slop	3, 4, 5, 7, 8, 9, 11, 12, 20, 24, 26, 27, 30, 33, 36, 37, 38, 39, 41, 46, 47, 48	3, 4, 6, 10, 17, 18, 19, 44, 45, 46
	Centroid	3, 4, 6, 7, 8, 9, 11, 12, 27, 38, 40, 46, 48	6, 10, 11, 17, 18, 44, 45, 46
PASAT	Mean	26, 37, 45, 48	4, 6, 17
	Integral	26, 37, 45, 48	4, 6, 17
	Slop	39, 40, 47	17, 18, 19
	Centroid	1, 28, 37, 39, 40, 42, 47	1, 4, 6, 17, 18, 19
Stroop C	Mean	4, 9, 25, 26, 35, 36, 44, 45, 46, 48	1, 3, 4, 6, 10, 17, 18, 19, 44, 45, 46
	Integral	4, 9, 25, 26, 35, 36, 42, 44, 45, 46, 48	1, 3, 4, 6, 10, 17, 18, 19, 44, 45, 46
	Slop	4, 7, 10, 13, 20, 21, 22, 25, 29, 32, 33, 35, 36, 37, 38, 39, 40, 41, 42, 43, 44, 45, 46, 47, 48	1, 2, 3, 4, 6, 9, 10, 17, 18, 19, 40, 45
	Centroid	3, 4, 9, 11, 12, 24, 29, 35, 36, 38, 42, 46, 48	1, 3, 4, 6, 10, 17, 18, 19, 44, 45, 46
TMT-B	Mean	5, 9, 11, 12, 16, 23, 25, 26, 27, 29, 37, 39, 41, 42, 43, 47, 48	4, 6, 10, 17, 18, 19, 44, 45, 46
	Integral	5, 9, 11, 12, 16, 23, 25, 26, 27, 29, 37, 39, 41, 42, 43, 47, 48	4, 6, 10, 17, 18, 19, 44, 45, 46
	Slop	9, 12, 25, 30, 38, 39, 41, 42, 43, 44, 45, 48	3, 4, 6, 10, 17, 19, 44, 45, 46
	Centroid	7, 11, 24, 36, 38, 39, 46, 48	10, 17, 18, 19, 45, 46

### Brain functional connectivity

**Fig. 4:**
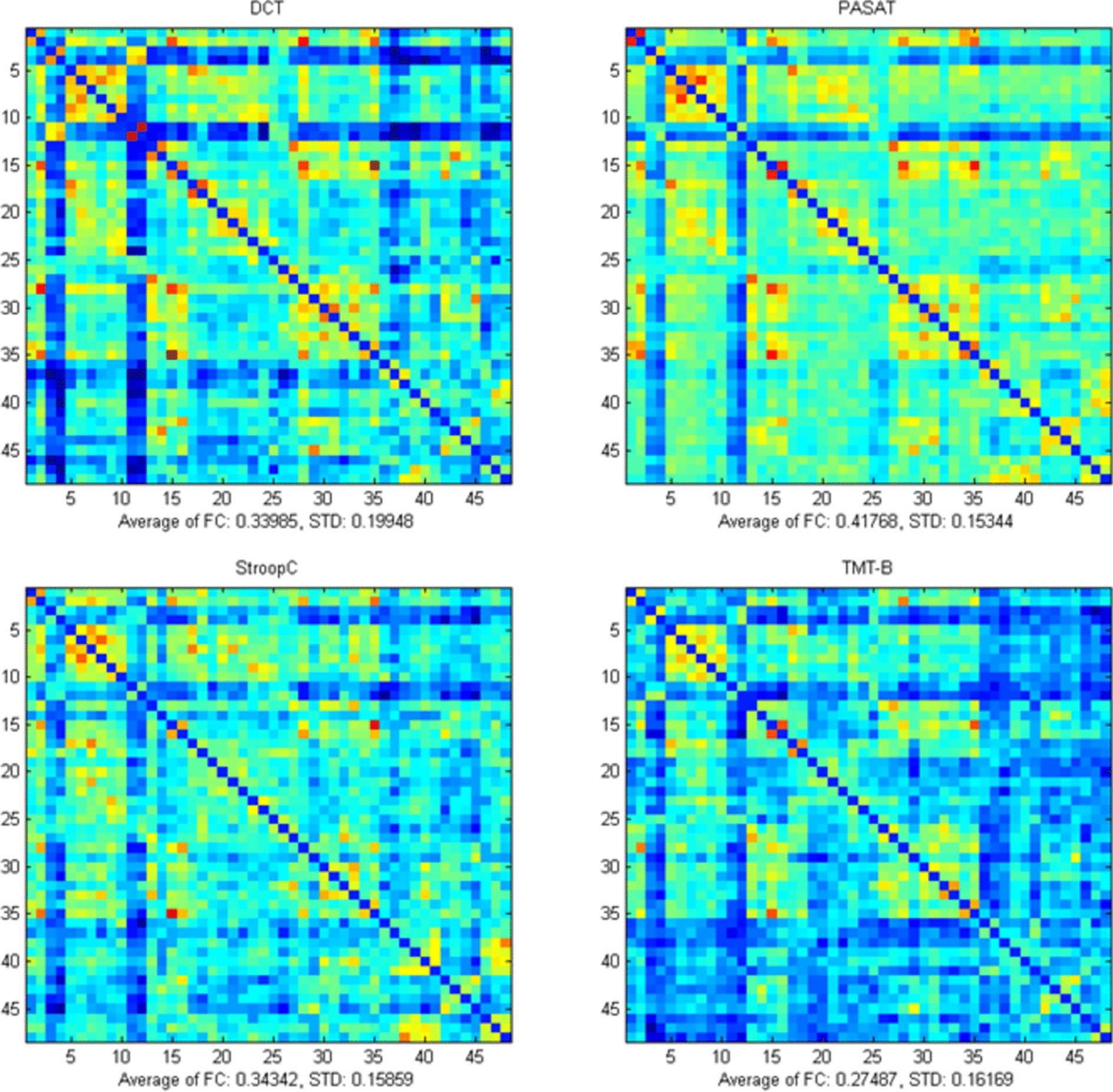
Functional connectivity matrix among the channels for the four attentional tasks

**Fig. 5:**
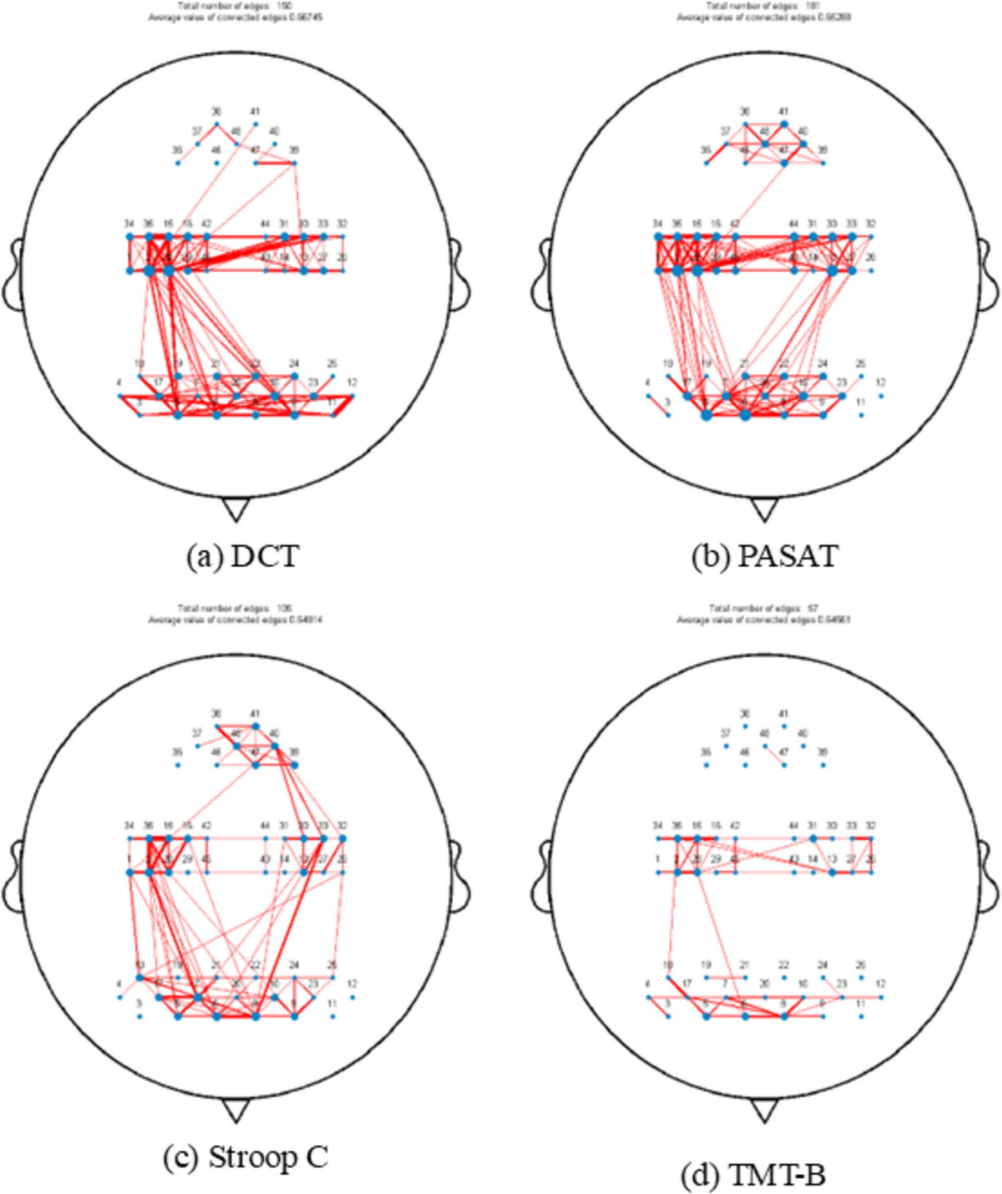
Functional connectivity exceeding the 0.5 threshold across four attention tasks. **a** DCT task; **b** PASAT task; **c** Stroop C task; **d** TMT-B task. The larger the blue circle is, the greater the number of connections will be, and the thicker the red line is, the stronger the connection will be

To gain an overarching perspective on functional connectivity across various states, we computed the average connectivity (derived from HbO) across all channels for all subjects. This process resulted in a 48 × 48 matrix representative of each attention task state, as illustrated in Fig. 4. To facilitate a clear visualization of connectivity differences, we established a threshold of 0.5 to generate functional connectivity maps for the distinct tasks. The size of the blue regions indicates the number of connections, while the thickness of the red lines signifies the strength of those connections. Notably, the frontal and right parietal lobes exhibited a higher density and strength of functional connections across all tasks. In the DCT task, the threshold for functional connections was exceeded by a total of 150 connections, which had a functional connectivity value of 0.33985 (SD: 0.19948). The PASAT task showed a higher total of connections, with 181 exceeding the threshold, and these connections had a functional connectivity value of 0.41768 (SD: 0.15344). The Stroop C task had 105 connections surpassing the threshold, with a functional connectivity value of 0.34342 (SD: 0.15859). The TMT-B task had the fewest connections above the threshold, with only 57, and these connections had a functional connectivity value of 0.27487 (SD: 0.16169) (Fig. 5).

**Fig. 6:**
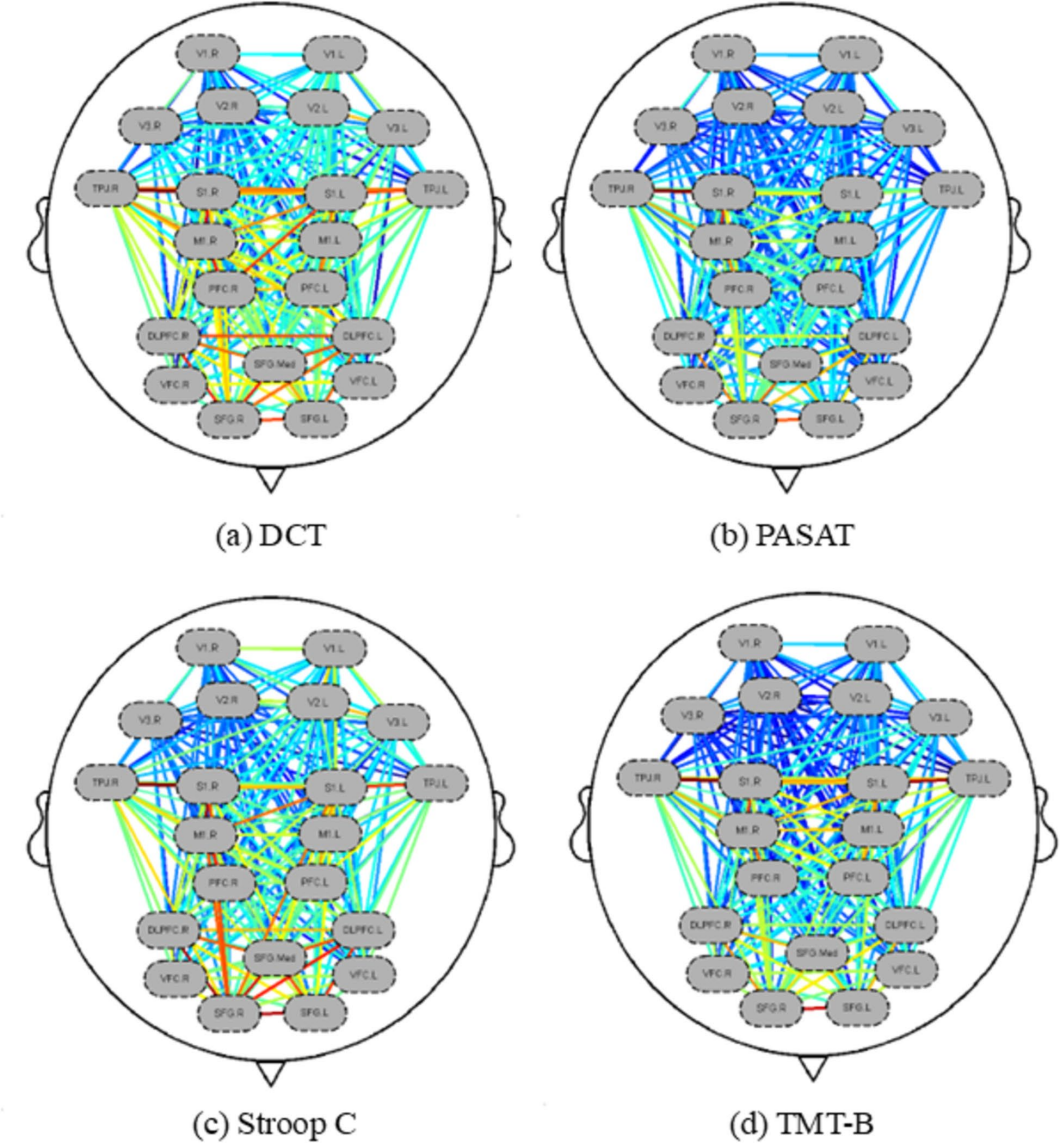
Four regions of interest (ROI) functional connections in attention tasks. **a** DCT task; **b** PASAT task; **c** Stroop C task; **d** TMT-B task. The redder the colour of the line, the stronger the functional connection

During the performance of the DCT task, a remarkable correlation in the fluctuations of HbO concentration was discerned among several brain regions. More precisely, such correlations were manifested between the frontal lobe, between the frontal lobe and the right parietal lobe, as well as between the bilateral parietal lobes. In the context of the PASAT task, the principal regions that warranted attention were the right frontal and parietal lobes. When it came to the Stroop C task, the emphasis was preponderantly placed on the bilateral frontal lobes, in addition to the connections both between the frontal and parietal lobes and those involving the occipital lobes. Conversely, the TMT-B task accentuated robust functional connectivity, principally within the bilateral frontal lobes and between the bilateral parietal lobes (Fig. 6).

Among the tasks, the divided attention task (PASAT) demonstrated a more intricate pattern of functional connectivity. To ascertain whether there were differences in functional connectivity between tasks, we conducted multiple comparisons, complemented by false discovery rate (FDR) correction. This approach allowed us to identify the differential connectivity channels between tasks. As depicted in Table 4, brain functional connectivity indeed varies with the nature of the task.

**Table 4:** ROI pairs with significant differences in connectivity strength between tasks (mean ± SD)

ROI to ROI	Connectivity strength		*F*	FDR-corrected *p*
	DCT	PASAT		
VFC.L ~ V1.R	− 0.07 ± 0.39	0.16 ± 0.24	4.048415	0.018302
VFC.L ~ V2.L	− 0.04 ± 0.44	0.24 ± 0.29	4.081449	0.019353
VFC.R ~ V1.R	− 0.04 ± 0.49	0.22 ± 0.30	4.129759	0.017638
VFC.R ~ V2.R	− 0.01 ± 0.34	0.28 ± 0.35	4.475312	0.005709

ROI to ROI	Connectivity strength		*F*	FDR-corrected *p*
	DCT	StroopC		
PFC.R ~ S1.L	0.79 ± 0.58	0.39 ± 0.49	5.464693	0.001541
PFC.R ~ TPJ.L	0.55 ± 0.60	0.20 ± 0.41	3.523854	0.011369
S1.L ~ V1.L	0.24 ± 0.43	0.48 ± 0.44	4.894543	0.023956
VFC.L ~ V1.R	− 0.07 ± 0.39	0.17 ± 0.43	4.048415	0.019926
VFC.L ~ V2.L	− 0.04 ± 0.44	0.24 ± 0.46	4.081449	0.016419
VFC.L ~ V3.L	− 0.03 ± 0.45	0.26 ± 0.51	3.205821	0.021056
VFC.R ~ V1.R	− 0.04 ± 0.49	0.22 ± 0.39	4.129759	0.017697

ROI to ROI	Connectivity strength		*F*	FDR-corrected *p*
	DCT	TMT-B		
M1.R ~ DLPFC.L	0.54 ± 0.51	0.32 ± 0.34	2.538477	0.030806
M1.R ~ DLPFC.R	0.52 ± 0.41	0.31 ± 0.31	2.65711	0.026403
DLPFC.L ~ DLPFC.R	0.78 ± 0.45	0.43 ± 0.37	4.302244	0.001996
DLPFC.L ~ SFG.Med	0.77 ± 0.58	0.43 ± 0.51	2.592273	0.033824
DLPFC.R ~ S1.R	0.55 ± 0.47	0.32 ± 0.37	2.692513	0.047552
PFC.R ~ S1.L	0.79 ± 0.58	0.45 ± 0.50	5.464693	0.009745
PFC.R ~ SFG.Med	0.49 ± 0.50	0.11 ± 0.48	4.595648	0.004086
PFC.R ~ V3.R	0.31 ± 0.45	0.05 ± 0.44	3.588644	0.022416
S1.L ~ V3.R	0.33 ± 0.47	0.02 ± 0.40	3.616365	0.020668
V2.L ~ V3.L	0.65 ± 0.77	0.27 ± 0.66	3.1142	0.022688

ROI to ROI	Connectivity strength		*F*	FDR-corrected *p*
	PASAT	StroopC		
DLPFC.R ~ V2.L	0.38 ± 0.36	0.13 ± 0.38	2.659942	0.046918
PFC.L ~ V2.R	0.28 ± 0.35	0.03 ± 0.37	2.699005	0.038108
PFC.R ~ SFG.Med	0.43 ± 0.34	0.11 ± 0.48	4.595648	0.023247
PFC.R ~ V3.R	0.29 ± 0.33	0.05 ± 0.44	3.588644	0.035156
S1.L ~ V3.R	0.27 ± 0.32	0.02 ± 0.40	3.616365	0.042395

ROI to ROI	Connectivity strength		*F*	FDR-corrected *p*
	StroopC	TMT-B		
S1.L ~ V1.L	0.48 ± 0.44	0.17 ± 0.35	4.894543	0.002871
VFC.R ~ V2.L	0.30 ± 0.38	0.05 ± 0.42	3.214309	0.048497
V2.R ~ V2.L	0.48 ± 0.82	0.09 ± 0.47	2.661066	0.036782

## Discussion

This study conducted a comparative analysis of activated cortical regions and functional connectivity under various task conditions, shedding light on the brain's mechanisms for executing these tasks. The results will serve as a valuable reference for subsequent research endeavours and clinical applications. Unlike many studies that focus on a specific mission, this investigation demonstrates that the patterns of cortical activation and functional connectivity vary among healthy middle-aged and older adults when engaging in a range of attention-demanding tasks. The comprehensive analysis disclosed that the collectively activated cortical regions across all missions encompassed the left BA-4, 17, which were consistently engaged in processing visual messages and executing tasks [20]. Our findings align with fMRI studies demonstrating parietal dominance in attention tasks [19].

The DCT, which measures sustained attention, activated the right prefrontal cortex, parietal sensory cortex, and occipital visual cortex, supporting Sarter's theory on the basal forebrain's role in'top-down'cognitive processes [21]. The visual cortex processed information, while the frontal and parietal lobes managed digit selection and elimination. Similar activation patterns were seen in the Stroop C and TMT-B tasks, particularly in brain areas BA-4, 6, 10, 17, 18, 19, 44, 45, and 46. BA-46 showed bilateral activation in DCT and TMT-B and unilateral left activation in Stroop C, suggesting left DLPFC's role in selective attention [22]. The PASAT showed modest brain activation, lacking significant activity in the dorsolateral prefrontal lobes, which diverges from the typical findings reported in most studies [23], possibly due to older participants with lower numerical skills. BA-10 was bilaterally activated in DCT and left-lateralized in Stroop C and TMT-B, indicating its role in prospective memory [24] and task-specific activation. Initial response speed was consistent across DCT, Stroop C, and TMT-B, with BA-2, 9 in Stroop C, and BA-3 in TMT-B showing significant speed. In PASAT, only the occipital visual cortex showed significant initial response speed. The observed activation of bilateral DLPFC during TMT-B and PASAT may reflect compensatory recruitment to counteract age-related declines in processing speed and working memory. This aligns with the Scaffolding Theory of Aging and Cognition (STAC) [25], which posits that older adults engage additional neural resources to maintain performance. Higher education levels in this study (52.9% with middle school education) may also enhance cognitive reserve, enabling efficient network reconfiguration [26]. Future studies should directly measure cognitive reserve proxies (e.g., education, occupational complexity) to validate this hypothesis.

Differences in functional connectivity across tasks may reflect the need for specificity in the type of attention. In this study's four attention-related tasks, the PASAT showed complex functional connectivity, while the TMT-B had a simpler profile. De Pasquale et al. discovered that in the damaged hemisphere, the dorsal attention and ventral attention, default mode, and frontoparietal executive network, as well as the cingulate cortex, exhibited a high degree of correlation with Bell's cancellation test scores following a stroke [27]. The PASAT task, which requires continuous auditory processing, working memory, and arithmetic operations, likely demands extensive coordination across distributed neural networks. The higher functional connectivity observed during PASAT (181 connections exceeding the threshold) may reflect the integration of frontal–parietal regions involved in maintaining attention, updating working memory, and performing calculations. The increased connectivity may represent a compensatory mechanism in middle-aged and elderly individuals to sustain divided attention under high cognitive load, as aging often necessitates greater neural resource mobilization [28]. Specifically, the right TPJ had strong connectivity with the right S1, related to matching sensory inputs with mental representations [29]. Bilateral frontal connections (Fig. 6c) in the selective attention task (Stroop C) may reflect the neural basis of conflict monitoring [30]. TMT-B task performance showed significant connectivity between bilateral SFG and between TPJ and S1. The SFG is part of the default mode network, associated with cognitive functions and attentional regulation [31] and the TPJ is related to the attentional shift orientation of the task [32]. No significant functional connectivity differences were found between PASAT and TMT-B. However, the DCT and PASAT tasks differed in connectivity strength involving the VFC and V1, V2, with PASAT requiring memory function activation and VFC's role in working memory. It has been demonstrated that the VFC is related to working memory [33]. The DCT showed stronger functional connections in the right PFC with the left S1 and TPJ compared to Stroop C, with TPJ's modulatory role in orienting networks being crucial for sustained attention in DCT [34]. Compared to TMT-B, DCT also had stronger connectivity between bilateral frontal lobes, which may support a “top-down” visual filtering mechanism [21]. The results of the present study are mostly consistent with the results of other neuroimaging studies. For instance, the Stroop task's bilateral frontal connectivity (Fig. 6c) mirrors EEG findings of increased frontal theta coherence during interference suppression [35]. However, discrepancies exist: the modest DLPFC activation during PASAT contrasts with fMRI studies showing robust prefrontal engagement [36], possibly due to fNIRS's limited sensitivity to deeper prefrontal regions. In addition, the strong TPJ-S1 connectivity in PASAT (Fig. 6b) parallels fMRI work linking TPJ to multisensory integration [29], but this has rarely been explored in fNIRS literature.

The results are unable to represent the entire situation, since fNIRS’s limited spatial resolution (~ 3 cm penetration depth) precludes imaging subcortical regions (e.g., thalamus) critical for attention [7]. Combining fNIRS with fMRI could enhance spatial specificity, as demonstrated by Andresen et al. [14], who hybridized fNIRS with fMRI to map deep and superficial hemodynamic responses. The thalamus and hippocampus, implicated in attentional gating and memory, were not assessed. Future studies should incorporate MRI to explore cortico-subcortical interactions, particularly in aging populations with known subcortical atrophy [27]. Moreover, attentional tasks encompass multiple aspects of cognition. That is to say, they pertain not only to the capacity for attention but also to short-term memory and computational abilities. For future studies, we can expand the sample size to explore differences between subjects of different genders or the presence of brain injury, etc.

## Conclusions

This research furnishes compelling evidence indicating that middle-aged and elderly individuals exhibit diverse patterns of brain activation and functional connectivity while engaging in various attentional tasks. This finding implies that the treatment of patients afflicted with attention disorders ought to be personalized and meticulously customized following the specific type and precise location of the impairment.

## Data Availability

No datasets were generated or analysed during the current study.
